# Haploidentical Donor Blood or Marrow Transplantation for Myelodysplastic/Myeloproliferative Overlap Neoplasms: Results from a North American Collaboration

**DOI:** 10.21203/rs.3.rs-2691216/v1

**Published:** 2023-03-21

**Authors:** Tania Jain, Hua-Ling Tsai, Hany Elmariah, Pankit Vachhani, Theodoros Karantanos, Sarah Wall, Lukasz Gondek, Asad Bashey, Alla Keyzner, Roni Tamari, Michael Grunwald, Sameem Abedin, Kalyan Nadiminti, Madiha Iqbal, Aaron Gerds, Auro Viswabandya, Shannon McCurdy, Monzr Al Malki, Ravi Varadhan, Haris Ali, Vikas Gupta, Richard John Jones, Salman Otoukesh

**Affiliations:** Sidney Kimmel Comprehensive Cancer Center at Johns Hopkins University; The Johns Hopkins University; H. Lee Moffitt Cancer Center and Research Institute; O’Neal Comprehensive Cancer Center, University of Alabama at Birmingham; The Ohio State University; Johns Hopkins Medicine; Northside Hospital; Icahn School of Medicine at Mount Sinai; Adult Bone Marrow Transplantation Service, Department of Medicine, Memorial Sloan Kettering Cancer Center; Levine Cancer Institute, Atrium Health; Medical College of Wisconsin; University of Wisconsin School of Medicine and Public Health; Mayo Clinic; Cleveland Clinic; Princess Margaret Cancer Centre; Hospital of the University of Pennsylvania; City of Hope National Medical Center; Johns Hopkins University School of Medicine; City of Hope; Princess Margaret Hospital; Sidney Kimmel Comprehensive Cancer Center; City of Hope

**Keywords:** haploidentical donor, transplantation, MDS/MPN-overlap

## Abstract

Haploidentical donors offer a potentially readily available donor, especially for non-White patients, for blood or marrow transplantation (BMT). In this collaboration across North America, we retrospectively analyzed outcomes of first BMT using haploidentical donor and posttransplantation cyclophosphamide (PTCy) in MDS/MPN-overlap neoplasms (MDS/MPN), an otherwise incurable hematological neoplasm. We included 120 patients, 38% of non-White/Caucasian ethnicity, across 15 centers with median age at BMT 62.5 years. The median follow-up is 2.4 years. Graft failure was reported in 6% patients. At 3-years, nonrelapse mortality (NRM) was 25%, relapse 27%, grade 3–4 acute graft versus host disease (GVHD) 12%, chronic GVHD requiring systemic immunosuppression 14%, progression-free survival (PFS) 48% and overall survival (OS) 56%. On multivariable analysis, statistically significant associations included older age at BMT (per decade increment) with NRM (sdHR 3.28, 95%CI 1.30–8.25), PFS (HR 1.98, 95% 1.13–3.45) and OS (HR 2.01, 95% CI 1.11–3.63), presence of mutation in EZH2/RUNX1/SETBP1 with relapse (sdHR 2.61, 95%CI 1.06–6.44), and splenomegaly at BMT/prior splenectomy with OS (HR 2.20, 95%CI 1.04–4.65). Haploidentical donors are a viable option for BMT in MDS/MPN, especially for those disproportionately represented in the unrelated donor registry. Disease-related factors including splenomegaly and high-risk mutations dominate outcomes following BMT.

## Introduction

Myelodysplastic/myeloproliferative overlap neoplasms (MDS/MPN) are a group of clonal myeloid neoplasms including the diagnoses of chronic myelomonocytic leukemia (CMML), MDS/MPN with neutrophilia (or atypical chronic myeloid leukemia or aCML per 2016 World Health Organization or WHO classification), MDS/MPN with *SF3B1* mutation and thrombocytosis, MDS/MPN, not otherwise specified (MDS/MPN-NOS).^1,2^ Over the years, several mostly small retrospective studies have established the curative potential of allogeneic blood or marrow transplantation (BMT) in MDS/MPN^3–6^, but none of these incorporated related HLA-haploidentical donors in a meaningful way. In the current era, the use of haploidentical donors in the broad scope of BMT has evolved significantly since the advent of post-transplantation cyclophosphamide (PTCy).^7–10^ The role of haploidentical donors in MDS/MPN offers the potential advantage of available donors in a timely manner for an otherwise incurable malignancy. Additionally, finding a fully matched donor in the donor registry can be challenging for non-White patients due to the lower diversity of donors from these populations. Therefore, haploidentical donors can often be suitable donor options for patients who may be ethnically underrepresented in the donor registry. On the other hand, theoretical concerns of delayed engraftment or graft failure have been raised with haploidentical donor BMTs owing to disease-related marrow fibrosis and splenomegaly. Hence, we conducted this study via our North American collaboration to systematically evaluate the clinical outcomes in MDS/MPN after haploidentical donor-PTCy BMT.

Genomic landscape plays a notable role in the prognostication of all MDS/MPN entities with worse prognoses attributed to higher number of mutations and specific high-risk mutations.^11–14^ Prior work has demonstrated that mutation(s) in *EZH2, RUNX1*, or *SETBP1* (*E*/*R*/*S*) is associated with a lower likelihood of response to hypomethylating agents, a commonly used non-transplantation therapeutic approach in MDS/MPN.^11^ Therefore, in this study, we sought to explore the role of genomic landscape, including a specific evaluation of *E*/*R*/*S* mutations, in determining outcomes of haploidentical donor BMT.

Since the prevalence of these diseases is low and morphological distinction for these individual entities is often obscure, we grouped the various MDS/MPN entities in this study. At the same time, features of dysplasia, as well as proliferation, remain a unifying feature of MDS/MPN entities. Furthermore, the prognosis of these individual entities especially with advanced or high-risk disease is poor, unless remission is achieved followed by consolidation with BMT.^11^

## Materials And Methods

### Patient selection and multi-institutional collaboration

This study leverages an ongoing multi-institutional collaboration of BMT centers across the USA and Canada to evaluate the role of BMT in rare myeloid malignancies. Fifteen institutions participated in this retrospective study, with Johns Hopkins University, Baltimore MD (USA) as the coordinating site. Each participating institution obtained approval from its respective institutional review board and data was transferred to Johns Hopkins University upon completion of data-sharing agreements with each participating site. The study was designed in keeping with the tenets of the Declaration of Helsinki. This study was designed prior to the publication of the 2022 update of WHO and International Consensus criteria definitions.^1,2^ Hence, the diagnosis of CMML, MDS/MPN with neutrophilia, MDS/MPN with SF3B1 mutation and thrombocytosis, and MDS/MPN-NOS was in accordance with the 2016 WHO classification for MDS/MPN.^15^ Bone marrow biopsy reports of all included patients were reviewed by the participating site investigator as well as the coordinating site investigator (T.J.) for adjudication of MDS/MPN diagnosis. Additional inclusion criteria for all centers were: (1) adult (age ≥18 years) patients who underwent a first BMT, (2) BMT using haploidentical donor defined as family donor mismatched for haplotype, and PTCy-based graft versus host disease (GVHD) platform, and (3) BMT timeline between January 2011 and December 2021. Patients who had a transformation to blast phase (>20% blasts in blood or marrow) at any point in the disease course and those who underwent haploidentical donor-cord blood BMT were excluded. All patients at all the collaborating institutions who met these criteria were included in the analysis.

### Denitions

Revised international prognostic scoring system (R-IPSS), clinical/molecular CMML-specific prognostic scoring system (CPSS-mol), and MDS/MPN responses to therapy were assessed as previously published.^16–18^ Spleen size was measured by imaging or physical exam. Given the variability of this measurement, we labeled spleen size of <12 cm on imaging or non-palpable on physical exam as normal, and ≥12 cm on imaging or palpable below costal margin on physical exam was considered enlarged. Time to neutrophil engraftment was defined as days from the day of BMT to the first of the three consecutive days when the absolute neutrophil count was ≥500/μL, while time to platelet engraftment was defined as days from the day of BMT to the first of the three consecutive data of platelets >20,000/ μL in the absence of platelet transfusions for 7 consecutive days.^19^ Graft failure was defined as a lack of donor hematopoietic cell engraftment following BMT (<5% donor chimerism) at any time following BMT, without evidence of disease relapse.^20^ Nonrelapse mortality (NRM) was death from any cause in the absence of disease relapse. Acute and chronic GVHD were graded per standard criteria.^21,22^ Day 0 of BMT was used as the reference day for time-to-event outcomes. Overall survival (OS) was defined from the date of BMT (day 0) to the date of death from any cause or censored at the last follow-up date for alive patients. The events of progression-free survival (PFS) included relapse or death, whichever occurred first.

### Cytogenetic and somatic mutation data

Cytogenetic results were deemed “high-risk” per those included in intermediate, high and very high-risk categories of R-IPSS.^17^ Next generation sequencing (NGS) was used to obtain somatic mutation data at individual participating institutions and results from these respective tests were used for analysis. NGS was obtained prior to BMT in all patients on whom the data is available, either at diagnosis or with the pre-BMT evaluations. High-risk mutations on NGS included mutations in *NRAS, SETBP1, RUNX1, EZH2, TP53, ASXL1, STAG2* as previously described.^13,14,23–25^ NGS was done at individual participating institutions and included commonly reported mutations in myeloid malignancies.

### Statistical analysis

For outcomes subject to competing events, cumulative incidences were reported and the distribution differences between groups were compared via Gray’s K-sample tests.^26^ When estimating the cumulative incidence function of relapse, NRM was the competing event and vice versa. When estimating the cumulative incidence of GVHD, the competing events included graft failure and death without graft-failure and without the corresponding GVHD event. OS and PFS were estimated via Kaplan-Meier method, and the distribution differences between groups were compared via log-rank test. Patients who did not relapse or die were censored on the date of last follow-up.

Cox proportional hazards model was applied in univariate and multivariable analyses to estimate the hazard ratio of OS and PFS.^27^ Fine-Gray subdistribution hazards model was used univariate and multivariable analyses of relapse, NRM, or GVHD outcomes.^28^ Covariates in multivariable analyses were selected based on clinical relevance and statistical significance noted on univariate analysis. All hypothesis testing was two-sided based on a significance level of 0.05 without considering multiplicity. Analyses were conducted in R version 4.2.2 (R Foundation for Statistical Computing, Vienna, Austria).

## Results

### Baseline patient and BMT details

**Figure 1:**
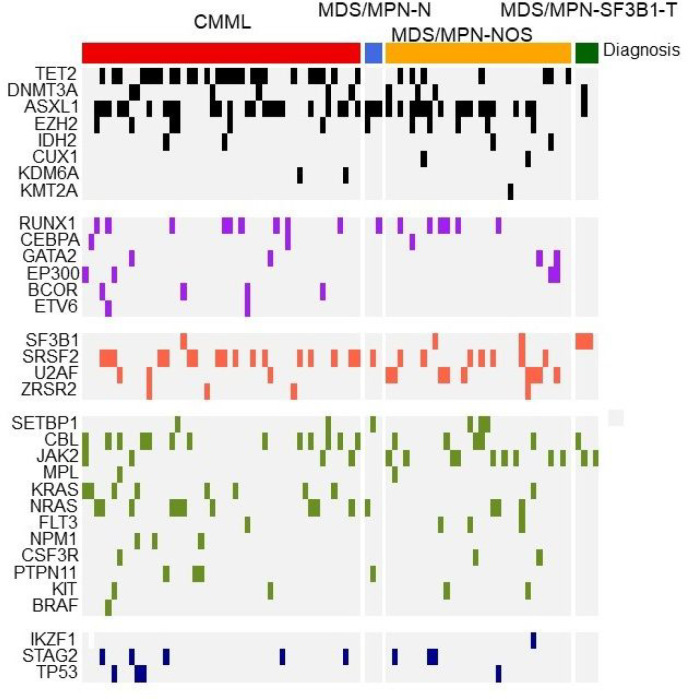
Mutational landscape of subset of patients undergoing haplo-BMT for MDS/MPN (n=90)

We identified 120 patients across the 15 participating institutions who underwent a first haplo-BMT for MDS/MPN using PTCy-based GVHD prophylaxis. A descriptive summary of these patients is shown in Table 1. Patients were more commonly of male sex (64%) and over one-third (37%) were ≥65 years of age, in keeping with the male predominance and older median age of diagnosis of MDS/MPN.^14,29^ Forty-six (38%) patients were of non-White/Caucasian race/ethnic background, who are disproportionately represented on the donor registry. Cytogenetic analysis revealed normal karyotype in 75 (63%) patients, as is often the case in MDS/MPN. NGS data was available in 90 patients and 45 (50%) had >3 mutations on NGS panel while 58 (64%) had one or more high-risk somatic mutations (Figure 1). As anticipated, bridging treatment prior to BMT varied significantly across all patients and hypomethylating agents were the most common agent for bridging. Seven (6%) patients had 10% or more blasts in the bone marrow at the time of BMT. Three patients had undergone splenectomy prior to BMT, two due to MDS/MPN, and one for a different malignancy.

### Clinical outcomes

**Figure 2:**
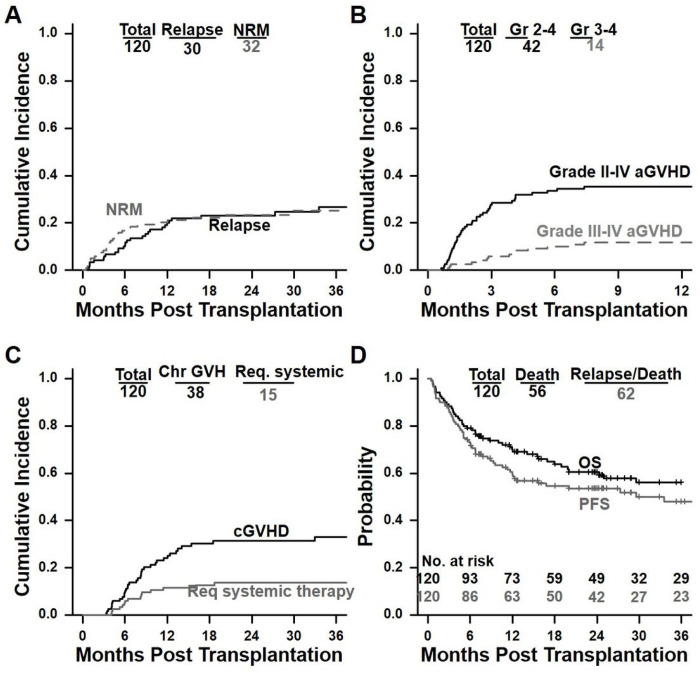
Clinical outcomes for entire cohort (N=120) (**2A**) Cumulative incidence of NRM and relapse; (**2B**) Acute GVHD grades 2–4 and grades 3–4; (**2C**) Chronic GVHD all grade and chronic GVHD requiring systemic immunosuppression; and (**2D**) Kaplan Meier estimates of OS and PFS

**Figure 3:**
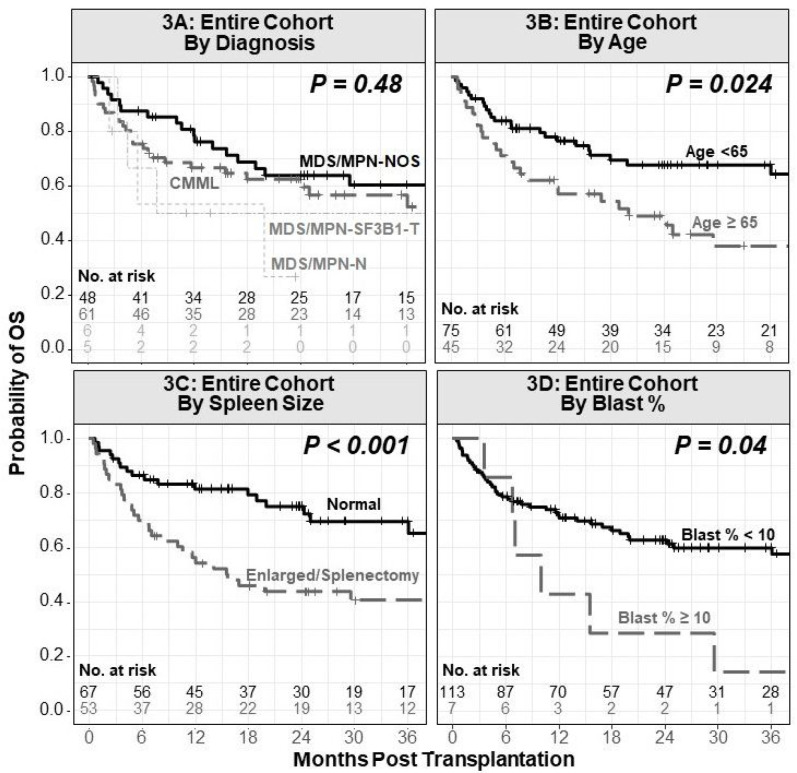
Difference in OS by (**3A**) individual diagnosis entities of CMML, MDS/MPN with neutrophilia (MDS/MPN-N), MDS/MPN-NOS, and MDS/MPN with SF3B1 mutation and thrombocytosis (MDS/MPN-SF3B1-T), (**3B**) patient age at BMT, (**3C**) spleen size at BMT, and (**3D**) bone marrow blast percentage in the total cohort of 120 patients

The median follow-up on this study is 2.4 years after BMT, based on the reverse Kaplan-Meier method. **Supplementary Table 1** summarizes engraftment, NRM, relapse, acute and chronic GHVD, PFS and overall survival OS. Figure 2 provides Kaplan-Meier analysis of all clinical outcomes. We evaluated OS and PFS for CMML, MDS/MPN-NOS, MDS/MPN with neutrophilia, and MDS/MPN with *SF3B1* mutations and thrombocytosis as separate entities and found no statistically significant difference (Figure 3A). Hence, we combined the 4 entities for the remaining analysis.

### Engraftment and outcomes after graft failure

Median time to neutrophil engraftment was 18 (interquartile range 16–22) days and platelet engraftment was 31 (interquartile range 22.5–41) days (**Supplementary Table 1**). Seven (6%) patients had graft failure, all of whom had received reduced intensity conditioning (RIC)/nonmyeloablative conditioning (NMAC), six had used a peripheral blood graft, and two received anti-thymocyte globulin and PTCy for GVHD prophylaxis. Three of these seven patients (43%) died before day +100 due to infections. The remaining four underwent a second BMT and two of those are alive at the last follow-up at day +481 and day +2337, respectively.

### NRM and Relapse

The cumulative incidence of NRM was 20% (95%CI 13–28%) at 1-year and 25% (95%CI 17–34%) at 3-years (Figure 2A and **Supplementary Table 1**). The cause of death among these patients were infection in 17 (14%), GVHD in 5 (4%), organ toxicity in 6 (5%), another malignancy in two (2%), and unknown in three patients (3%). The cumulative incidence of relapse was 20% (95% CI 13–27%) at 1-year and 27% (95%CI 18–36%) at 3-years. In total, 30 (25%) patients had relapsed of whom 24 (20%) had died as a result of relapsed disease, by the last follow-up. Seven patients underwent donor lymphocyte infusion (DLI), most commonly for molecular relapse, of whom two restored full donor chimerism while one had only a transient improvement in chimerism. Four patients underwent a second BMT after relapse, two of whom had received a prior DLI also. All four of these patients are deceased at the last follow-up from persistent disease.

### Acute and chronic GVHD

Most patients who experienced acute GVHD had highest grade of grade 2. At 1-year, the cumulative incidence of acute GVHD grade 2–4 was 35% (95%CI 27–44%) and of grade 3–4 acute GVHD was 12% (95%CI 6–18%) (Figure 2B and **Supplementary Table 1**). The skin and gut were the most commonly involved organs in 27/42 (64%) patients. The cumulative incidence of chronic GVHD at 3 years was 33% (95%CI 24–42%) and of chronic GVHD requiring systemic therapy was 14% (95%CI 7–20%) (Figure 2C and **Supplementary Table 1**).

### PFS and OS

At 1-year and 3-years, the probability of PFS was 60% (95%CI 51–69%) and 48% (95%CI 39–59%) and the probability of OS was 70% (95%CI 62–79%) and 56% (95%CI 47–67%), respectively, as tabulated in **Supplementary Table 1.** The respective Kaplan-Meier curves for PFS and OS are shown in Figure 2D.

### Univariate analysis

**Figure 4:**
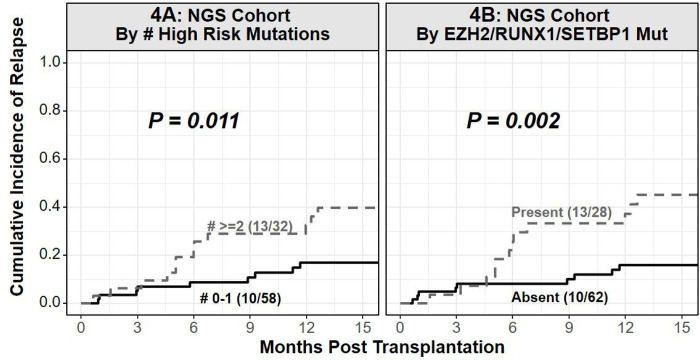
Difference in relapse by (**4A**) number of high-risk mutations, and (**4B**) *EZH2, RUNX1,* or *SETBP1* mutations in the cohort of 90 patients with NGS data

The univariate analysis including patient, disease, and BMT variables for NRM, relapse, PFS, and OS is detailed in Table 2. In the univariate analysis for OS, older patient age at BMT (HR 1.53 per decade increase in age, 95%CI 1.07–2.18, *P*=0.02, Figure 3B), year of BMT prior to 2019 (HR 0.44, 95%CI 0.23–0.82, *P*=0.01), splenomegaly at BMT/prior splenectomy (HR 2.57, 95%CI 1.48–4.44, *P*<0.005, Figure 3C), ≥10% blasts in marrow at BMT (HR 2.38, 95%CI 1.01–5.59, *P*=0.046, Figure 3D), and RIC/NMAC (HR 2.44, 95%CI 1.04–5.71, P=0.04) were associated with inferior OS. The presence of ≥2 high-risk mutations (sdHR 2.70, 95%CI 1.20–6.05, *P*=0.02, Figure 4A), and *E*/*R*/*S* mutations (sdHR 3.33, 95%CI 1.47–7.51, *P*<0.005, Figure 4B), were associated with a significantly higher risk of relapse following BMT.

### Multivariable analysis

Patient age at BMT, year of BMT, RIPSS, presence of *E*/*R*/*S* mutation, splenomegaly at BMT, donor age, and intensity of conditioning regimen were included in the multivariable analysis. Blast percentage was not included in the multivariable analysis because only seven patients had blasts over 10%. A complete multivariable analysis is shown in Table 3. Older age at BMT (sdHR 3.28 for every 10 years increment in age, 95%CI 1.30–8.25, *P*=0.01) was associated with higher NRM. RIC/NMAC and presence of *E*/*R*/*S* were significantly associated with higher relapse rate (sdHR 3.90, 95%CI 1.32–11.49, *P*=0.01 for RIC/NAMC and HR 2.61, 95%CI 1.06–6.44, *P*=0.04 for *E*/*R*/*S* mutations).

Inferior PFS and OS were noted with older age at BMT (HR 1.98, 95%CI 1.13–3.45, *P*=0.02 for PFS and HR 2.01, 95%CI 1.11–3.63, *P*=0.02 for OS), and splenomegaly at BMT or a splenectomy prior to BMT (HR 1.78, 95%CI 0.91–3.51, *P*=0.09 for PFS and HR 2.20, 95%CI 1.04–4.65, *P*<0.04 for OS). As a result of the counterpoise of lower NRM and higher relapse, conditioning intensity did not show a significant association with PFS or OS (HR 1.51, 95%CI 0.75–3.05, *P*=0.25 for PFS and HR 1.20, 95%CI 0.57–2.52, *P*=0.64 for OS). Notably, the choice of myeloablative conditioning, over RIC/NMAC, was statistically significantly correlated with younger age at BMT in this analysis, corroborating observations from clinical practice (**Supplementary Figure 1**).

## Discussion

Our study provides a comprehensive description of the outcomes of haplo-BMT in MDS/MPN in a cohort of 120 patients in this multi-institutional collaboration. The potentially curative role of BMT in high-risk CMML was recently elucidated in comparison to non-BMT options.^5^ We demonstrate that haploidentical donors can be used with BMT outcomes similar to what has been historically reported with matched donors, in the rare diagnosis of MDS/MPN. This is particularly important for populations who are less likely to find a fully matched donor in the unrelated donor registry. Graft failure rate was under 10% and OS was 70% at 1-year and 56% at 3 years in our study. In the recent international analysis of CMML patients without AML transformation, BMT resulted in OS of about 30–35% at 3 years, with a majority (~75%) of donors being HLA-matched siblings or unrelated donors.^5^ Japanese nationwide registry data reported an OS of 48.5% at 3-years in MDS/MPN-NOS, using a variety of related, unrelated, and cord blood donors.^30^ Notably, 40% patients in this study were under 50 years of age at BMT. In a Mayo Clinic cohort of 17 CMML and 8 MDS/MPN-NOS patients without antecedent blast transformation, BMT with matched donors resulted in a graft failure of 6% and 0%, and OS of 47% and 41%, at 2-years, respectively.^6^ Among 14 patients with MDS/MPN with neutrophilia, the Japanese registry study reported 54% OS at 1-year, using predominantly matched donors and select cord blood donors.^3^ While the timeline of BMT in all these studies varies, outcomes in our study are comparable to the limited reports presented above, underscoring that donor availability should not preclude consideration of BMT for patients with MDS/MPN whose outcomes remain poor in the absence of the BMT.

This study also explores modifiable disease-related features, in the form of spleen size control and blast reduction, which can possibly be optimized prior to BMT to allow for superior disease control and survival following BMT. We previously demonstrated the role of enlarged spleen size in negatively impacting relapse outcomes in Myelofibrosis.^8,31^ JAK inhibitors have shown meaningful spleen size reduction in Myelofibrosis^32–34^ and have an emerging role in the management of CMML by targeting JAK-STAT dependent GM-CSF signaling in CMML.^35,36^ Hence, JAK inhibitors may address spleen size reduction prior to BMT in MDS/MPN as in Myelofibrosis, an evaluation warranted in future studies. While MDS/MPN (except CMML) were not included in the pivotal VIALE-A trial, retrospective studies have demonstrated disease control with a combination of hypomethylating agents with BCL-2 inhibitor, venetoclax, in select patients with elevated blasts in MDS/MPN.^37^ We cannot identify an optimal bridging therapy in this study due to the variable availability of drugs over the years and various factors guiding bridging therapy selection in the real-world, including individual center practices. However, a systematic evaluation of the role of JAK inhibitors and BCL-2 inhibitors for disease control and as a bridge to BMT in MDS/MPN is warranted.

Intensity of conditioning regimen is often a matter of discussion in planning BMT, especially in chronic myeloid malignancies where average age of diagnosis or BMT if often over 60 years. As is noted in our study, decisions on conditioning intensity are commonly driven by age and the comorbidity status of an individual patient. In Myelofibrosis, MDS and other myeloid malignancies, retrospective studies of higher intensity conditioning demonstrate possibility of better disease control but at the expense of higher NRM^38–41^, similar to what we note in this study. Ultimately, overall survival is not statistically different. Age and comorbidities are key in determining the intensity of conditioning chemotherapy, in that younger or fitter patients are enriched in the myeloablative cohort, as also noted in our study. Hence, patient selection remains a critical confounder to consider when interpreting the role of intensity of conditioning in a retrospective manner.

A growing body of evidence has uncovered the role of the genomic landscape in the overall prognosis as well as response to hypomethylating agent therapy in MDS/MPN.^11,13,42^ Our study further elaborates on the role of somatic mutations in MDS/MPN, including previously defined high-risk mutations, in prognosticating outcomes of haploidentical donor BMT. In two cohorts of CMML patients, mutations in *ASXL1, CBL*, *RUNX1*, *NRAS*, and *SETBP1* were associated with adverse survival.^16,42^ We also demonstrated higher prevalence of high-risk mutations, specifically *EZH2,* in men with MDS/MPN, which may be responsible for inferior overall outcomes when compared to women.^14^ E/R/S, and *ASXL1* mutations have also been associated with a lower risk of response to non-BMT therapy, especially hypomethylating agents, in MDS/MPN.^11,43^ In the context of BMT, somatic mutation data has historically been limited to CMML. Mutations in *DNMT3A, TP53, ASXL1*, and *NRAS* correlated with inferior survival in two different studies.^12,44^ The presence of high-risk mutations, specifically *E*/*R*/*S*, or the presence of ≥2 high-risk mutations significantly increased the risk of relapse. Collectively, these data suggest that MDS/MPN harboring high-risk mutations identified in the non-transplantation context also in uence relapse following BMT.

MDS/MPN is a rare, yet consequential, disease entity. This extensive report of haploidentical donor BMT in MDS/MPN was feasible due to our robust multi-institutional collaboration. The present analysis nevertheless remains limited by its retrospective nature and variability of practice across centers. We combined the four entities within MDS/MPN for this analysis given the high-risk nature of all four entities when considered for BMT and because our initial analysis demonstrated no statistical difference in OS among CMML, MDS/MPN-NOS, MDS/MPN with neutrophilia, and MDS/MPN with *SF3B1* mutation and thrombocytosis. The strategy of combining diseases for analyses increases the sample size but introduces the confounding effect of variable nuances of these entities.

## Conclusion

We demonstrate feasibility and comparable outcomes with haplo-BMT and PTCy in MDS/MPN in a multi-institution study with reference to previously published data. Given the otherwise incurable diagnosis of MDS/MPN, this study provides rational for expanding potential donor pool to include haploidentical donors in patients undergoing transplant evaluation. Optimization of disease-related factors such as spleen size and blast percentage reduction prior to BMT should be explored in future studies. Mutation landscape associated with MDS/MPN can guide outcomes with BMT. Future studies are needed to explore pre-BMT therapies and their on modifying mutational burden and post-transplantation outcomes.

## Figures and Tables

**Table 1:** Baseline patient, disease and BMT details (N=120)

Characteristic		n (%) or median (range) [N=120]

***Patient details***		

Median age at diagnosis in years (range)		61 (17–74)

Median age at BMT in years (range)		62.5 (18–75)

Median months from diagnosis to BMT (range)		10.35 (1.1 – 399.2)

Age at BMT ≥65 years		45 (37.5%)

Male sex		77 (64.2%)

Race/ethnicity:	Caucasian	74 (61.7%)
	African American	21 (17.5%)
	Hispanic	13 (10.8%)
	Asian	11 (9.2%)
	Alaskan Native	1 (0.8%)

Diagnosis:	CMML	61 (50.8%)
	MDS/MPN-NOS	48 (40.0%)
	MDS/MPN with neutrophilia	5 (4.2%)
	MDS/MPN with SF3B1 and thrombocytosis	6 (5.0%)

KPS at BMT:	70–80	46 (38.8%)
	90–100	74 (61.6%)

Comorbidity index:	0–1	65 (54.2%)
	≥2	55 (45.8%)

***Disease-related details***		

High-risk cytogenetics*		25 (20.8%)

Mutations on the NGS panel (total n=90):	0	3 (3.3%)
	1–3	42 (46.7%)
	>3	45 (50%)

Number of high-risk mutations on NGS (total n=90)	0	32 (35.6%)
	1	26 (28.9%)
	≥2	32 (35.6%)

Mutations in *E/R/S* present		28 (31.1%)

R-IPSS risk category:	Very low/ Low	48 (40%)
	Intermediate/High/Very high	72 (60%)

CPSS-Mol risk category (for patients with CMML, n=61)	Low	7 (11.5%)
	Intermediate-1	15 (24.6%)
	Intermediate-2	22 (36.1%)
	High	14 (22.9%)
	Missing	3 (4.9%)

Bridging therapy prior to BMT:	None	14 (11.7%)
	Hydrea only	5 (4.2%)
	HMA/JAK inhibitor/immunomodulatory^¥^	82 (68.3%)
	Induction (including HMA venetoclax)^£^	19 (15.8%)

CR/PR with bridging therapy prior to BMT (total n=106):		40 (37.7%)

Spleen size at BMT:	Normal	67 (55.8%)
	Enlarged	50 (41.7%)
	Splenectomy	3 (2.5%)

Marrow blasts at BMT:	<10%	113 (94.2%)
	≥10%	7 (5.8%)

***BMT-related details***		

BMT year:	2011–2018	63 (52.5%)
	2019–2021	57 (47.5%)

Recipient CMV seropostive		80 (66.7%)

Donor age at BMT in years:	<30	33 (27.5%)
	30–45	62 (51.7%)
	>45	25 (20.8%)

Male donor gender		73 (60.8%)

ABO mismatch:	None	78 (65.0%)
	Minor	15 (12.5%)
	Major	26 (21.7%)
	Bidirectional	1 (0.8%)

Conditioning regimen intensity:	Myeloablative	22 (18.3%)
	Reduced intensity	51 (42.5%)
	Non-myeloablative	47 (39.2%)

GVHD prophylaxis (with PTCy):	Tacrolimus MMF	97 (80.8%)
	Sirolimus MMF	19 (15.8%)
	ATG-Cyclosporin	4 (3.3%)

Graft source:	Bone marrow	25 (20.8%)
	Peripheral blood	95 (79.2%)

Median CD34+ cell dose (range)		5 (0.86 – 23.8) ×10^6^/kg

* Per R-IPSS, del(7q), +8, +19, i(17q), −7, inv(3)/t(3q)/del(3q), double including −7/del(7q), Complex: 3 or more abnormalities.

¥ Regardless of prior hydrea use

£ Regardless of prior HMA, JAK inhibitor or immunomodulatory agent use

**Table 2:** Univariate analysis for NRM, relapse, DFS, and OS (N=120)

	NRM		Relapse		PFS		OS	

	sdHR (95% CI)	*P*	sdHR (95% CI)	*P*	HR (95% CI)	*P*	HR (95% CI)	*P*

Age at BMT (10-year increment)	1.40(0.88–2.23)	0.15	1.51(1.00–2.28)	0.05	1.63(1.15–2.02)	**0.006**	1.53(1.07–2.18)	**0.02**

Sex (female vs male)	0.80(0.38–1.67)	0.55	0.72(0.34–1.55)	0.40	0.70(0.41–1.19)	0.19	0.72(0.41–1.27)	0.25

Race/ethnicity:								
Caucasian	Ref		Ref		Ref		Ref	
African American	0.75 (0.26–2.16)	0.59	1.30 (0.52–3.24)	0.57	1.04 (0.51–2.11)	0.91	0.96 (0.44–2.09)	0.92
Hispanic/Asian/Alaskan Native American	1.58 (0.73–3.42)	0.24	1.20 (0.51–2.80)	0.68	1.53 (0.84–2.79)	0.16	1.70 (0.92–3.11)	0.09

Years from diagnosis to BMT	1.08(1.02–1.14)	**0.01**	0.86(0.70–1.05)	0.15	1.03(0.96–1.11)	0.39	1.05(0.97–1.13)	0.21

BMT year (2019–2021 vs 2011–2018)	0.41(0.18–0.92)	**0.04**	0.64(0.30–1.38)	0.26	0.47(0.27–0.82)	**0.008**	0.44(0.23–0.82)	**0.01**

Diagnosis:								
CMML	Ref		Ref		Ref		Ref	
MDS/MPN-NOS	1.30(0.62–2.71)	0.48	0.93(0.46–1.90)	0.85	1.06(0.62–1.81)	0.82	0.94(0.54–1.66)	0.84
MDS/MPN-N or MDS/MPN-SF3B1-T	2.98(1.07–8.34)	**0.04**	0.33(0.05–2.27)	0.26	1.48(0.61–3.59)	0.39	1.84(0.75–4.51)	0.18

KPS (≥90 vs <90)	0.96(0.48–1.93)	0.91	0.50(0.25–1.02)	0.056	0.65(0.39–1.07)	0.09	0.83(0.48–1.41)	0.48

HCT CI (≥2 vs 0–1)	1.32(0.67–2.62)	0.42	0.88(0.43–1.78)	0.72	1.07(0.65–1.76)	0.79	1.16(0.69–1.96)	0.58

RIPSS (intermediate/high/very high vs very low/low)	1.84(0.85–3.98)	0.12	0.94(0.46–1.95)	0.88	1.42(0.82–2.43)	0.21	1.40(0.79–2.46)	0.25

Bridging therapy:								
None	Ref		Ref		Ref		Ref	
Hydrea only	2.14(0.32–14.16)	0.43	No relapse		0.93(0.19–4.63)	0.93	1.15(0.22–5.99)	0.86
HMA/ JAK inhibitor/IMiD	1.16(0.36–3.73)	0.80	1.35(0.42–4.33)	0.61	1.33(0.56–3.14)	0.51	1.38(0.54–3.53)	0.50
Induction	1.58(0.46–5.40)	0.46	1.32(0.32–5.42)	0.70	1.51(0.57–4.05)	0.41	1.55(0.54–4.49)	0.42

CR/PR prior to BMT	0.79 (0.38–1.63)	0.52	1.88 (0.81–4.34)	0.14	1.20 (0.69–2.09)	0.52	1.27 (0.71–2.27)	0.43

Spleen size at BMT (enlarged/splenectomy vs normal)	2.10(1.04–4.25)	**0.04**	1.68(0.82–3.45)	0.15	2.17(1.31–3.61)	**<0.005**	2.57(1.48–4.44)	**<0.001**

Marrow blasts at BMT (≥10% vs >10%)	1.60(0.55–4.70)	0.39	1.86(0.58–5.95)	0.29	1.97(0.84–4.60)	0.12	2.38(1.01–5.59)	**0.047**

High-risk cytogenetics (presence vs absence)	1.06(0.47–2.41)	0.88	0.68(0.27–1.73)	0.42	0.83(0.44–1.56)	0.57	0.90(0.47–1.75)	0.76

High-risk NGS (presence vs absence)	0.70(0.29–1.72)	0.44	2.65(0.87–8.12)	0.09	1.34(0.69–2.63)	0.39	0.99(0.49–1.98)	0.98

Number of high-risk mutations (≥2 v 0–1)	0.61(0.23–1.60)	0.32	2.70(1.20–6.05)	**0.02**	1.51(0.81–2.83)	0.19	1.23(0.63–2.41)	0.54

*E/R/S* mutation (presence vs absence)	0.56(0.19–1.60)	0.28	3.33(1.47–7.52)	**<0.005**	1.72(0.92–3.23)	0.09	1.18(0.59–2.34)	0.64

Donor age (10-year increment)	1.20(0.88–1.63)	0.25	1.12(0.84–1.50)	0.45	1.21(0.98–1.49)	0.07	1.21(0.97–1.51)	0.09

Graft source (blood vs marrow)	0.85(0.37–1.95)	0.70	0.91(0.38–2.14)	0.82	0.85(0.47–1.55)	0.60	0.87(0.46–1.65)	0.68

GVHD prophylaxis:								
Tacrolimus	Ref		Ref		Ref		Ref	
Sirolimus	0.36(0.08–1.62)	0.18	2.02(0.95–4.33)	0.07	1.07(0.54–2.11)	0.85	0.87(0.41–1.85)	0.71
ATG cyclosporine	4.16(1.29–13.48)	**0.02**	No relapse	-	2.07(0.64–6.68)	0.22	2.26(0.70–7.33)	0.17

Conditioning intensity:								
MAC	Ref		Ref		Ref		Ref	
RIC/NMAC	1.77(0.70–4.49)	0.23	3.42(0.81–14.39)	0.09	2.90(1.35–6.76)	**0.01**	2.44(1.04–5.71)	**0.04**

CMV reactivation requiring intervention	1.37(0.69–2.68)	0.37	0.43(0.16–1.15)	0.09	0.76(0.43–1.33)	0.33	0.87(0.49–1.54)	0.63

ATG, anti-thymocyte globulin; CMV, cytomegalovirus; CR/PR, complete response/ partial response; HCT-CI, hematopoietic cell transplant comorbidity index; HMA, hypomethylating agent; IMiD, Immunomodulatory drugs; JAK, Janus kinase; KPS, Karnofsky performance score; MDS/MPN-N, MDS/MPN with neutrophilia; MDS/MPN-SF3B1-T, MDS/MPN with SF3B1 mutation and thrombocytosis; NGS, next generation sequencing;

**Table 3:** Multivariable analysis for NRM, relapse, DFS, and OS (n=90)

	NRM		Relapse		PFS		OS	
	sdHR (95% CI)	*P*	sdHR (95% CI)	*P*	HR (95% CI)	*P*	HR (95% CI)	*P*
Age at BMT (10-year increment)	3.28(1.30–8.25)	**0.01**	1.11(0.67–1.81)	0.69	1.98(1.13–3.45)	**0.02**	2.01(1.11–3.63)	**0.02**
BMT year (2019–2021 v 2011–2018)	0.39(0.10–1.47)	0.16	0.71(0.28–1.80)	0.48	0.55(0.28–1.10)	0.09	0.53(0.25–1.13)	0.10
RIPSS (intermediate/high/very high v very low/low)	2.53(0.84–7.68)	0.10	0.82(0.32–2.12)	0.68	1.44(0.74–2.81)	0.28	1.35(0.66–2.75)	0.42
*E/R/S* mutation (presence vs absence)	0.43(0.14–1.33)	0.14	2.61(1.06–6.44)	**0.04**	1.24(0.64–2.40)	0.52	0.88(0.42–1.82)	0.73
Spleen size at BMT (enlarged/splenectomy v normal)	1.19(0.34–4.14)	0.78	1.70(0.74–3.87)	0.21	1.78(0.91–3.51)	0.09	2.20(1.04–4.65)	**0.04**
Donor age (10-year increment)	1.36(0.87–2.12)	0.18	1.09(0.68–1.77)	0.71	1.17(0.85–1.63)	0.34	1.14(0.79–1.62)	0.49
Conditioning intensity (RIC/NMAC v MAC)	0.37(0.12–1.16)	0.09	3.90(1.32–11.49)	**0.01**	1.51(0.75–3.05)	0.25	1.20(0.57–2.52)	0.64

## Data Availability

Data dictionary, study protocol, and statistical analysis plan will be available with publication. Deidentified patient data will be made available upon a reasonable request to the corresponding author after publication.

## Abbreviations

BMT: Blood or marrow transplantation
DLI: Donor lymphocyte infusion
*E*/*R*/*S*: *EZH2*/*RUNX1*/*SETBP1*
GVHD: Graft versus host disease
HR: Hazard ratio
MDS/MPN: Myelodysplastic/myeloproliferative overlap neoplasm
MDS/MPN-NOS: MDS/MPN not otherwise specified
NGS: Next generation sequencing
NMAC: Non-myeloablative conditioning
NRM: Nonrelapse mortality
OS: Overall survival
PFS: Progression-free survival
PTCy: Posttransplantation cyclophosphamide
RIC: Reduced intensity conditioning
R-IPSS: Revised international prognostic scoring system
sdHR: Subdistribution hazard ratio
